# Diseases of the Heart and Thoracic Aorta

**Published:** 1884-10

**Authors:** Robert H. Babcock


### BOOK REVIEWS.

---

DISEASES OF THE HEART AND THORACIC AORTA. *By* BYRON BRAMWELL, M.D., F.R.C.P. *Lecturer on the Principles and Practice of Medicine, and on Practical Medicine and Medical Diagnosis in the Extra-Academical School of Medicine, Edinburgh; Pathologist to the Edinburgh Royal Infirmary; Additional Examiner in Clinical Medicine in the University of Edinburgh. Late Physician and Pathologist to the New-castle-on-Tyne Infirmary; Formerly Medical Officer to the Tynemouth Union Workhouse Hospital, The Prudhoe Memorial Convalescent Home, The Tyne Floating Hospital, Etc., Etc. With six illustrations. New York:* D. APPLETON & CO., *Bond Street. 1884.*

This work, with its neat binding, exceptionally good print, its numerous beautiful illustrations, is very attractive, and in its comparative freedom from typographical errors forms a pleasing contrast to the publications of another New York house, whose well-known name ought to be a guarantee of good work. Although we noted but four errors of the compositor, two of them are grave and ought not to have escaped the eye of the proof-reader. There are several other errors which would appear to have existed in the author's manuscript, and are not inexcusable. On page 69, in speaking of Cheyne-Stokes respiration, he says: * * * "It only occurs in advanced cases of cardiac disease." As he elsewhere speaks of its occurrence in other, such as cerebral affections, his meaning here evidently is that it occurs only in advanced cases of cardiac disease. Again, in the note on page 137, he states: "The para-sternal line is an imaginary line drawn vertically downwards over the front of the chest, midway between the left border of the sternum and the left nipple." This displays not only that laxity of expression, which runs through the entire work, but exemplifies as well prolixity and tautology. The sentence quoted should read: The left para-sternal line is an imaginary one drawn vertically midway between the left border of the sternum and the left nipple. On page 157, in discussing the reduplication of the first heart-sound, he says: "In order that the first sound may be perceptibly reduplicated—*i. e.*, that the mitral and tricuspid first sounds may be separated by a perceptible interval—it is necessary to have a considerable degree of asynchronism between the closure and tension of the aortic and pulmonary valve flaps—a more considerable asynchronism than is required to produce perceptible doubling of the second sound." The valves concerned in the production of the first sound are the mitral and tricuspid, not the semilunar; hence the words "aortic and pulmonary" should be replaced by *mitral* and *tricuspid*. In a note on page 203 occur the words, "an abnormal accentuation of blood behind the mitral orifice." The word "accentuation" has here been inadvertently substituted for accumulation. Yet it is perhaps more likely that this is the fault of the compositor than of the author. On page 397, the expression "liquid ammoniæ" is used in place of liquor ammoniæ or liquid ammonia. The author's prolixity and tautology, to which we have alluded, are so flagrant throughout as to become painfully wearisome. In consequence, our enjoyment of the work was destroyed, and its perusal grew to be a tiresome task. If carefully revised and pruned, the book might be curtailed a fourth, we presume, and rendered far more readable. The author states, in his preface, that the context has been taken almost unchanged from his lectures—which may account for many of the repetitions. The reiteration of *heads* and various "steps" is well enough when students are taking notes of a lecture, but it mars the symmetry and enjoyment of a work that is meant to be classical.

We regret, also, to observe certain expressions which, although employed quite generally by English medical writers, seem to us objectionable. We refer to such as the following, on page 319: "The left apex-beat." What is the "left apex-beat" if it is not the apex-beat? We do not recognize a right apex-beat. However, passing from faults of style to that most important part, the subject-matter of the book, we have much to commend and some things to deplore. Chapters one and two (which are introductory) are excellent, and will elucidate many points that are so often obscure to the student of medicine. The discussion of Cheyne-Stokes respiration is good, and the author's attempt to explain the phenomena as due to "irritable weakness" of the respiratory center (page 74) is not only original, but has much to recommend it to thoughtful consideration as meeting the needs of the case as fully as those of Traube, Sansom, or Filehne. The discussion of the several theories advanced in explanation of the systolic murmur heard in the pulmonary area in many cases of anæmia, is also highly instructive and interesting. Throughout the work the pathology of the various diseases discussed, is excellent and not so detailed as to weary the student and ultimately render his ideas on the subject misty. In general, the affections treated are handled well and clearly but, like most English writers on diseases of the heart and vascular system, he is inclined, we think, to include too many doubtful facts in the etiology and to assert them dogmatically. Thus, on page 373, he states: "In fact, it would appear that anæmia is a predisposing cause of acute rheumatism." He is also inclined to the opinion (page 569) that "cirrhosis of the kidney and cirrhosis, or fibroid degeneration of the heart, are both due to chronic alcoholism." He often speaks of syphilis as producing acute endocarditis, and, on page 692, he enumerates "strain (more especially frequently-repeated and sudden increases of the blood pressure), syphilis, alcoholic excesses, gout, rheumatism, exposure to cold and depressing influences of all kinds," as the "great causes" of chronic endarteritis. German pathologists express themselves much more guardedly on these points, because not regarding them as substantiated, and we think their position more scientific. It is of immense satisfaction to be able to trace the cause of a given affection so explicitly, but it seems to us somewhat hasty.

In his remarks on pericarditis with effusion, the author certainly conveys the impression that the friction sounds obtain undiminished and unaltered even in the height of the effusion. Friction sounds are undoubtedly audible in many cases with considerable liquid in the pericardium, but it is likewise true that many times they disappear with the distension of the sac; and where they remain, their intensity and location is very apt to change. We are glad to have him, on pages 319–320, call attention to the anatomical fact (as stated by Duchek) of great clinical significance, that the triangular shape of the area of cardiac dullness in pronounced pericardial effusion, without pleuritic adhesion, is due to the retraction of the borders of the lungs, rather than to the shape of the pericardial sac; and that hence a greatly enlarged heart may also push aside the edges of the lungs and simulate an extensive pericardial effusion. The faintness of the heart-sounds in such a case serves but to mislead further. *Apropos* of this matter, we once saw Ziemssen deceived by exactly such a condition. The autopsy alone revealed the source of the error.

Altogether, this work is to be highly recommended, and, though not contributing much of originality, is instructive and likely to form a valuable addition to the library of the general practitioner as well as of the tyro, in the clinical study of the diseases of which it treats. Doubtless many will be glad to find an elucidation of the cardiograph and sphygmograph with a plentitude of normal and abnormal tracings. The 317 admirable illustrations would be valuable of themselves independently of the context.

ROBERT H. BABCOCK, M. D.

